# Study of the Genetic Etiology of Primary Ovarian Insufficiency: FMR1 Gene

**DOI:** 10.3390/genes7120123

**Published:** 2016-12-13

**Authors:** Maitane Barasoain, Gorka Barrenetxea, Iratxe Huerta, Mercedes Télez, Begoña Criado, Isabel Arrieta

**Affiliations:** 1 Department of Genetics, Physical Anthropology and Animal physiology, Faculty of Science and Technology, University of the Basque Country, Bilbao, 48940, Spain; maitane.bhf@gmail.com (M.B.); iratxe.huerta@medikosta.com (I.H.); mertxe.telez@medikosta.com (M.T.); 2 Department of Medical-Surgical Specialities, Faculty of Medicine, University of the Basque Country, Bilbao, 48940, Spain; gbarrenetxea@reproduccionbilbao.es; 3 Reproduction Bilbao, Bilbao, 48014, Spain; 4 Genetic Counseling and Prenatal Diagnosis Laboratory GENETIC, Bilbao, 48014, Spain; 5 Cooperativa de Ensino Superior Politecnico e Universitario (CESPU), Porto, 4760-409, Portugal; mbegona.criado@ipsn.cespu.pt

**Keywords:** FMR1, POI, menopause

## Abstract

Menopause is a period of women’s life characterized by the cessation of menses in a definitive way. The mean age for menopause is approximately 51 years. Primary ovarian insufficiency (POI) refers to ovarian dysfunction defined as irregular menses and elevated gonadotrophin levels before or at the age of 40 years. The etiology of POI is unknown but several genes have been reported as being of significance. The *fragile X mental retardation 1* gene (*FMR1*) is one of the most important genes associated with POI. The *FMR1* gene contains a highly polymorphic CGG repeat in the 5′ untranslated region of exon 1. Four allelic forms have been defined with respect to CGG repeat length and instability during transmission. Normal (5–44 CGG) alleles are usually transmitted from parent to offspring in a stable manner. The full mutation form consists of over 200 repeats, which induces hypermethylation of the *FMR1* gene promoter and the subsequent silencing of the gene, associated with Fragile X Syndrome (FXS). Finally, *FMR1* intermediate (45–54 CGG) and premutation (55–200 CGG) alleles have been principally associated with two phenotypes, fragile X tremor ataxia syndrome (FXTAS) and fragile X primary ovarian insufficiency (FXPOI).

## 1. Menopause

The reproductive capacity of a woman depends principally on two factors, the number of follicles in the ovaries during the reproductive years and quality of oocytes within those follicles. The number of follicles is determined genetically before birth, during the embryonic development and it is termed ovarian follicular reserve. Only a few hundreds of these follicles complete their development, while most of them become atretic and are eliminated during the recruitment.

The initial recruitment and the resulting loss of follicles begin during the fetal stage and continue throughout the lifespan. When the ovarian reserve is below a certain threshold, some reproductive events occur, such as irregular menses, and finally this process ends in menopause. Menopause coincides with a recount of a few hundred follicles stored in the ovaries [1].

Menopause is a period of women’s life characterized by cessation of menses in a definitive manner. It occurs approximately at 50.7 years [2], however it ranges between 48 and 54 years. With respect to this this range, there is a differentiation between early menopause (40–44 years), late menopause (>55 years) and premature menopause (<40 years). The origin of menopause resides in the follicular depletion of the ovaries. The appearance of menopause can be natural, when it occurs gradually and progressively by the normal ageing of the ovary, or artificial, caused by the removal of the ovaries with or without hysterectomy or by radiotherapy or chemotherapy [3].

Ovaries age through quick depletion of follicles and consequently of oocytes. This process begins during the embryonic stage and extends to menopause. Usually, a healthy female possesses ~400,00 primordial follicles at the beginning of puberty and of these only 400–500 reach maturity during the reproductive life span of an adult female [4].

Depletion of the ovarian reserve implies a parallel growth of luteinizing hormone (LH) and follicle-stimulating hormone (FSH) [5]. There is also a fall in anti-Müllerian hormone (AMH) because of a reduction of the antral follicle pool. This occurs parallel to the reduction of the primordial follicles pool.

The diagnosis of menopause is principally clinical, after one year of amenorrhea. The hormones analyzed for the diagnosis are primarily FSH, whose levels should be higher than 40 IU/L, and estradiol, with values below 30–50 pg/mL. Currently, AMH levels are considered an adequate marker of follicular depletion. This hormone varies minimally during the cycle and its fall to undetectable levels could be a signal of the start of the last stage of menopausal transition [3].

## 2. Primary Ovarian Insufficiency

Primary ovarian insufficiency (POI) is defined as the loss of the ovarian hormonal function in a woman before or at the age of 40 years [6]. This term was suggested by Welt [7] and three subtypes are differentiated:

- Occult: normal levels of gonadotrophins, reduced fecundity and regular menses.
- Biochemical: elevated levels of gonadotrophins, reduced fecundity and regular menses.
- Overt: elevated levels of gonadotrophins, reduced fecundity and irregular or absent menses.

There has been controversy with the term POI. Previously, the term premature ovarian failure (POF) was used and it was defined as cessation of menstrual cycles before the age of 40 years with deficiency of sexual hormones and elevated levels of gonadotrophins [8]. The use of POF suggests an irreversible failure of the ovary but in some cases ovarian failure is not permanent: Fifty percent of women with POI present intermittent ovarian function and 5%–10% conceive spontaneously [9,10]. In these cases, the use of the term POF is not entirely adequate and consequently the term POI was proposed.

The common symptom among women with POI is reduced fertility, but premature estrogen deficiency has other clinical consequences including higher risk of osteoporosis or mortality increase due to cardiovascular causes [11]. In addition, women suffering a premature menopause have higher risk of problems such as anxiety, depression and other psychological symptoms compared to women with normal ovarian function [12,13,14].

### Etiology of POI

The etiology of POI is unknown, but some mechanisms have been considered, including a reduction in the number of oocytes or ovarian reserve, an increase in follicle destruction and alterations in the follicular recruitment and maturation.

Although the majority of POI cases are idiopathic, there are three principal causes for the development of this pathology: chromosomal abnormalities, genetic causes and autoimmune alterations [15]. With lower implication, metabolic defects and other causes have been related to POI.

**a. Chromosomal abnormalities:**

The X chromosome includes important genes for determining ovarian function. Numerical or structural changes in the X chromosome, including Turner’s syndrome (45, X), triple X syndrome (47, XXX) and deletions or translocations in this chromosome, have been associated with POI, as reviewed by Fortuño and Labarta [16].

**b. Genetic causes:**

A high number of candidate genes associated with POI have been analyzed (Table 1). The majority of them are related to different processes of the follicular maturation and many of them have their locus in the X chromosome. These are some of the most important genes related with POI, although only the association of *fragile X mental retardation 1* (*FMR1*) and *bone morphogenetic protein 15* (*BMP15*) genes has been thoroughly demonstrated:

- *FMR1* gene. It has its locus in Xq27.3 and many studies propose that premutation of the *FMR1* gene is the cause of approximately 6% of POI cases [1,17,18,19].
- *FMR2* gene. It is also denominated AF4/FMR2 Family Member 2 (AFF2) and is localized in Xq28. Murray et al. in 1998 [20] found microdeletions in this gene as the cause of the 1.5% of the analyzed POI women.
- BMP15, localized in Xp11.22, is part of the transforming growth factor β (TFG-β) family involved in cellular processes that occur during embryonic development and tissue formation. BMP15 protein is expressed specifically in the oocyte and its expression maintains high levels during follicular maturation and ovulation [21]. Mutations of this gene in mice and sheep have been identified that give rise to altered ovulation [21,22].
- *Growth differentiation factor 9* (GDF9), localized in 5q31.1, encodes a member of the transforming growth factor expressed in the oocyte and it plays its role in differentiation of the own oocyte and granulosa and thecal cells. Mutations in single nucleotide polymorphisms (SNPs) of this gene have been identified among patients with POI [23].
- *Luteinizing hormone receptor* (*LHR*) and *follicle-stimulating hormone receptor* (*FSHR*) are localized in chromosome 2, in *loci* 2p21 and 2p21-p16 respectively. They encode receptors for gonadotropic hormones FSH and LH. These hormones are essential for the right function of menstrual activity. Both regulate the production of sex steroid hormones, estradiol and progesterone, by thecal and granulosa cells rounding the growing follicle. Alterations in these receptors could reduce the ability of these receptors to join these hormones, decreasing its activity.
- Recently, *stromal antigen 3* (*STAG3*) gene, in 7q22.1, has also been associated with POI. This gene encodes a subunit of the cohesin ring specific of meiosis and it is also connected with azoospermia [24,25].

**c. Autoimmune alterations:**

Autoimmune mechanisms are the cause in 14.3% of POI cases [26]. Associated autoimmune disorders are, among others, Addison’s disease, dry-eye syndrome or Sjógren’s syndrome, autoimmune polyglandular syndrome, rheumatoid arthritis, or systemic lupus erythematosus [27].

Some antibodies have been investigated as serological markers of ovarian immunity, such as 3β-hydroxysteroid dehydrogenase (3β-HSD) autoantibodies, antibodies against FSH and LH receptors, anti-thyroid antibodies, autoantibodies against luteal body, pellucid zone or oocytes [28]. Goswami et al. [29] assessed the prevalence of thyroid peroxidase autoantibodies in patients with POI and they showed that 24% of POI cases presented with these antibodies. However, none of these antibodies is considered valid as serological marker that can confirm a diagnostic of autoimmune POI.

**d. Metabolic and other causes:**

Some inherited enzymatic pathway disorders have been associated with ovarian follicular dysfunction leading to POI, as reviewed by Cox and Liu [30].

Pelvic surgery or cancer treatment could lead to POI. Radiotherapy and chemotherapy are strongly related to the development of this pathology. The effect of these treatments in the loss of ovarian function depend on dose, patient’s ovarian reserve and age [9].

## 3. *FMR1* Gene

*FMR1* gene was identified in 1991 [31,32,33] and coincides with the fragile site FRAXA localized in Xq27.3.

### 3.1. Characteristics of the Gene

The *FMR1* gene is 40 kb in length and contains 17 exons. The ~4 kb full length mRNA [34] codes for a protein with a maximum length of 632 amino acids and a molecular mass of 69 kDa called *fragile mental retardation protein* (FMRP).

The gene contains a highly polymorphic CGG repeat in the 5′ untranslated region (UTR) of the exon 1. It shows also a CpG island located 250 bp upstream the repeat [35].

### 3.2. Alleles

The CGG repeat sequence is polymorphic in length and four alleles are differentiated:

- Normal: It ranges from 5-4 CGG repeats with a mode in 30 repeats
- Intermediate or grey zone: this class includes alleles with sizes at the high end of normal range, from 45 to 54 repeats. These alleles are found in 5%–9% of the general population [36,37,38].
- Premutation: Alleles with repeat sizes from 55 to 200 repeats. The prevalence of premutation alleles in the general population is estimated to be 1:130–250 among women and 1:250–810 among men [39,40,41].
- Mutation: It includes alleles with more than 200 CGG repeats. A prevalence is estimated of 1:4000 men and approximately 1:5000–8000 women [42,43].

There is no consensus about the estimated prevalence of *FMR1* alleles and this prevalence varies between geographical areas and the population assessed [41,44,45].

### 3.3. Transcriptional Regulation

Among carriers of normal, intermediate and premutation alleles, *FMR1* gene is transcriptionally active and FMRP is produced. However, when the number of repeats is over 200, cytosines of the CGG sequence and the ones of the upstream CpG island are methylated, leading to the silencing of the gene and the resulting absence of FMRP [46]. The absence of this protein is the principal cause of fragile X syndrome (FXS).

The epigenetic phenomenon of methylation occurs in the 5′ position of cytosines and induces, on one hand, the condensation of the chromatin and, on the other hand, it prevents the binding of transcription factors such as upstream stimulatory factor (USF)1, USF2 and nuclear respiratory factor (NRF)-1.

Changes in histones H3 and H4 have been also described. These histones are associated with the 5′UTR region of the *FMR1* gene and normally they are acetylated in their lysine residues. In mutated cells this acetylation is reduced which suggests that methyl CpG binding proteins recruit histone deacetylases which in turn induce chromatin condensation and prevent the transcription machinery from binding [47]. It has been observed that H3 shows lysine 4 demethylated and lysine 9 methylated [48].

In vitro treatment of mutated hypermethylated cells with methylation inhibitors, such as 5-azadeoxycytidine (5-aza-dC), partially reactivates genetic expression, demonstrating that methylation is the cause of genetic inactivation [49]. Normally, active *FMR1* gene has an open chromatin structure but when CGG repeat expansion occurs it leads to deacetylation and methylation of the CGG repeat and its promotor resulting in a packed chromatin, and causing the inactivation of the gene. Treatment of fragile X cell lines with 5-aza-dC produces demethylation and acetylation so that chromatin is open and transcription is partially recovered [50].

### 3.4. Instability of CGG Repeat

Characterization of *FMR1* gene brings to light a new type of mutation mechanism based in expansion of microsatellite sequences. According to Pearson et al. [51], the repeat mutation process is dynamic, with products that continue to mutate within tissues and across generations. The initial change in a microsatellite locus increases the probability of subsequent changes towards mutation. Therefore, longer sequences are more likely to undergo an expansion mutation than shorter ones. Moreover, length of the repeat sequence correlates with disease severity and age of onset: the more repeats, the more severe the disease and the earlier its onset. This is what is known as genetic anticipation [51,52].

Several pathologies such as Huntington´s disease, myotonic dystrophy or Kennedy’s disease are caused by trinucleotide expansions. These expansions can result in two mechanisms, loss or gain of function. Fragile X syndrome is an example of loss-of-function mechanism in which the gene product is either not produced or produced at lower levels. When there is a gain of function, mRNA or protein attain a new cellular function depending on the localization of the expansion in the gene [52].

There are some risk factors influencing the instability of *FMR1* CGG repeat during transmission:

- Number of repeats: The risk of expansion increases with the length of the repeat. Normal alleles are usually transmitted from parent to offspring in a stable manner. Intermediate alleles can be unstable upon transmission, leading to a full mutation in several generations. However, some authors reported an expansion of an intermediate allele to a full mutation in only two generations [53,54,55]. Premutation alleles are associated to a high risk of expansion to full mutation in one generation [56].
- Sex of the transmitting parent: Among normal and intermediate alleles, transmission from a male is less stable than from a female. However, when a premutation allele is transmitted by a female, CGG repeat suffers an expansion in almost all cases, although not always to full mutation. Moreover, among females, instability is at the same time proportional to the length of the repeat, so that alleles with more CGGs have a higher risk of expansion [56,57]. When premutation is transmitted by a male, the CGG repeat can expand, contract or remain unchanged, with the risk of expansion to full mutation being infrequent.
- Purity of the repeat: CGG repeat is not pure; it is interrupted by an AGG trinucleotide. In the general population, CGG repeats are interrupted every 9–10 repeats [(CGG)_9_AGG(CGG)_9_AGG(CGG)_n_] [58]. Normal and intermediate alleles have usually two interruptions [59], whereas premutation alleles tend to have one or no AGGs at the 5′ end of the repeat [60,61,62]. The length of pure CGG repeats is also important. In 1994, Kunst and Warren [63] established that alleles with more than 24 pure CGG repeats will be unstably transmitted. More recently, it has been shown that there is a strong association between number of AGG interruptions and stability of the repeat [64,65].
- “*cis*” elements: “*cis*” elements with the *FMR1* gene can affect the stability of the repeat. The more studied are flanking microsatellites and (SNPs). Mutated alleles are in linkage disequilibrium with flanking microsatellite markers, like DXS548 and FRAXAC1 [66]. It has also been suggested that SNPs can proportionate an advance in the study of CGG repeat instability. A research from Brightwell et al. in 2002 [67] noted that association between SNP ATL1 (rs4949:A>G) and flanking microsatellite markers reflect the mutational history of CGG repeat expansion. Previous investigations of our group do not support this suggestion [68].

### 3.5. FMRP

FMRP is expressed preferentially in brain and testis. It has an important role in the regulation of protein synthesis in dendrites of the neurons [69], necessary for learning and memory [70].

FMRP is an RNA binding protein whose subcellular distribution is principally cytoplasmic. However, some isoforms are localized in the nucleus, which is consistent with the nuclear localization (NLS) and exportation (NES) signals of the protein. This indicates that FMRP shuttles between nucleus and cytoplasm [71].

The best characterized motifs are the ones responsible of its mRNA binding: two *K homology* (KH) domains in exons 8 and 10, an RGG box (arginine-glycine-arginine) in exon 15 [72] and an RNA binding domain in the amino terminal region of the protein [73]. The RGG box binds to a G-quartet structure present in several mRNAs associated with FMRP. At the same time, KH domains are important for the interaction between FMRP and polyribosomes.

FMRP associates with polyribosomes of the endoplasmic reticulum and with free ribosomes in the cytoplasm in an RNA-dependent manner via ribonucleoprotein particles (RNP). These RNPs contain proteins such as FXR1P and FXR2P, homologs to FMRP, nucleolin, Y-box Binding Protein 1 (YB-1), Nuclear FMRP Interacting Protein 1 (NUFIP1), Cytoplasmic FMRP Interacting Protein 1 (CYFIP1) and Cytoplasmic FMRP Interacting Protein 2 (CYFIP2) [50]. As a consequence of this association, FMRP abolishes the translation of a specific group of mRNAs [74]. Several works have identified some of these RNAs and have shown that these RNAs encode for proteins with an important role in neuronal function, synaptic plasticity and neuronal maturation [75,76].

Synaptic plasticity is essential for memory and learning and includes the long-term potentiation (LTP) and long-term depression (LTD). Synaptic plasticity is expressed at a pre- and post-synaptic level. At a post-synaptic level, LTD can be produced by the activation of *N*-methyl-d-aspartic acid receptors (NMDA) or metabotropic glutamate receptors (mGluR). At the same time, this activation induces the internalization of AMPA receptors, necessary for LTD, and the expression of FMRP [77] and other proteins.

According to the denominated “metabotropic glutamate receptors (mGluR) theory of fragile X syndrome” proposed by Bear et al. in 2004 [78]: as a consequence of the synthesis of FMRP, this protein acts as an inhibitor of the translation of certain proteins that reduce the internalization of α-amino-3-hydroxy-5-methyl-4-isoxazolepropionic acid (AMPA) receptors. When FMRP is absent, like in FXS individuals, this internalization increases resulting in an excess of LTD [69].

Some works in FMR1 knock-out mice have shown that the use of glutamate 5 receptor (mGluR5) antagonists, such as 2-methyl-6-(phenylethynyl)pyridine (MPEP), reduces the internalization of AMPA receptors caused by FMRP absence [79].

### 3.6. Associated Pathologies

*FMR1* gene is associated principally with three pathologies: FXS, tremor ataxia syndrome (FXTAS) and primary ovarian insufficiency (FXPOI).

FXS etiology is basically CGG trinucleotide repeat expansion in the 5′UTR region of *FMR1* gene. It has been proposed that the *FMR1* gene is uniquely responsible for FXS [71]. Premutated alleles, of between 55 and 200 CGG repeats, were not associated with any pathology until recently but nowadays they are clearly related to FXPOI and FXTAS. Intermediate or grey zone alleles, between 35 and 54 CGG repeats, are also related to the development of these two pathologies.

Presence of phenotypic characteristics associated with premutation alleles led to analysis of *FMR1* expression. Research of Tassone et al. [80,81] and Tassone and Hagerman [82] showed that *FMR1*-mRNA levels are increased approximately fivefold within premutation carriers (from two to ten times the normal), although protein expression is slightly reduced. In 2011, Tassone et al. [83] determined that in these premutation alleles there are different transcriptional start sites and polyadenylation sites leading to the different expression levels of these alleles with respect to normal ones. This, together with the fact that permutation-associated pathologies do not appear among individuals with FXS, denotes that the mechanism of these pathologies is different from that of FXS.

**a. Fragile X Syndrome**

Identification of Fragile X Syndrome:

In 1943 James Martin and Julia Bell [84] published the first pedigree showing a mental retardation associated to X chromosome. It was termed Martin-Bell syndrome. In 1974 Lehrke [85] estimated that 25%–50% of mental retardation could be attributed to mutations in the X chromosome. This estimation, while too high, established a new concept; X chromosome-linked mental retardation (XLMR).

In 1969 Lubs [86] detected a secondary constriction at the end of the long arm of a chromosome from group C in four mentally retarded males and three obligate carrier females of the same family. The chromosome was identified later by autoradiography as the X chromosome. The constriction, which served as a cytogenetic marker, was localized later in Xq27.3 [87], corresponding with the fragile site FRAXA. Richards et al. [88] reanalyzed the Martin-Bell pedigree and found that in affected males a cytogenetic variation was expressed as described previously by Lubs, which they defined as a fragile site. In that moment the syndrome was denominated fragile X syndrome (OMIM #300264).

Existence of a cytogenetic marker allowed the identification of a large number of families with the X chromosome marker, revealing the syndromic nature of this disorder. But analysis of these families pointed to an inheritance pattern more complex than a X-linked recessive inheritance pattern. There were affected heterozygous females and unaffected males that transmitted the cytogenetic marker to their daughters. In 1984 this phenomenon was denominated Sherman’s paradox [89] and its origin was not resolved until 1991, when the *FMR1* gene responsible of the syndrome was identified.

Fragile X Mutation:

The principal cause leading to variability in CGG repeat number is thought to be slipped-strand mispairing during DNA replication: when a new strand is synthesizing, the template strand, due to its repetitive nature, folds and form a secondary structure, so that DNA polymerase dissociates temporally from the template strand and re-associates to one or several previous repeat units, creating an extra loop of repetitive DNA that, if not repaired, would generate an expansion [47]. In 1994, Richards and Sutherland [90] proposed that this process would occur specifically in the Okazaki fragments.

Over the last years, cases of individuals carrying the full mutation but with a non-methylated CGG repeat and promoter have been described [91]. These individuals are phenotypically normal, proving that is the methylation and not the repeat expansion which causes the fragile X phenotype. There are also individuals whose mutation is methylated only in a percentage of its cells (methylation mosaicism) and that show a protein expression corresponding to non-methylated cells [92].

Moreover, more than 15 deletions, ranging from 1.6 kb to 13 mb, affecting part or the entire *FMR1* gene have been described, leading to FMRP absence and, therefore, generating the fragile X phenotype [93]. There are also point mutations leading to this phenotype that do not cause FMRP absence [94,95,96]. For example, I304N mutation (replacement of an isoleucine by an asparagine in aminoacid 304) in the second KH domain leads to a FMRP protein that does not associate with polyribosomes [94].

Fragile X Phenotype:

FXS is the most common known cause of inherited intellectual disability and autism. Epidemiological studies estimate that prevalence of this syndrome in the general population is 1:2500–1:8000 females and approximately 1:5000 males [40,41,97].

It includes a wide range of clinical, cognitive and behavioral dysfunctions. Clinical features include a long and narrow face with a prominent jaw and forehead, large ears, unusually flexible fingers, flat feet, and enlarged testicles (macroorchidism) after puberty in males. At a cognitive level, 80%–90% of males with FXS present mild to moderate intellectual deficiency, with an important attention deficit and delayed development of speech and language. At a behavioral level, hyperactivity is characteristic, and one-third of individuals with FXS have autism [98,99]. Of these, 18%–36% meet the criteria for autism disorder (AD) and 43%–67% present an autistic spectrum disorder (ASD).

**b. Fragile X-Associated Tremor/Ataxia Syndrome**

FXTAS (OMIM #300263) was firstly described in 2001 by Hagerman, Leehey et al. [100] in older (50-60 years) male carriers of a premutation allele. These authors defined it as a multisystem neurological disorder with tremor and ataxia as principal features.

The presence of intranuclear inclusions along with clinical characteristics constitutes the key factor for FXTAS. These inclusions are widely distributed along the brain, principally in the hippocampus and amygdala [101,102]. Inclusions have been described in carrier males with alleles as small as 70 CGG repeats. They are ubiquitin positive and they have been localized in neurons and astrocytes. Number and size of inclusions increase with age, which is related with the progressive nature of the syndrome [103]. Number of inclusions is also related with CGG repeat size [102,104].

At a molecular level, an RNA toxic-gain-of-function mechanism has been proposed [105]. *FMR1* transcripts with expanded CGG repeat are incorporated in a messenger ribonucleoprotein (mRNP) particle and translocated out of the nucleus. These transcripts do not join to the 40S ribosomal subunit and consequently translation is affected, reducing the levels of FMRP in the nerve cell. In response to lowered FMRP levels, an increase in transcription factors is produced, leading to a growth of *FMR1* transcription and therefore to an increase in *FMR1*-mRNA levels [59].

Alternatively, *FMR1* transcripts with long CGG repeats may sequester high quantities of CGG-binding proteins resulting also in increased *FMR1* transcription. Nerve cells attempt to eliminate the raised *FMR1* transcripts by the use of chaperones and components of the ubiquitin–proteasome degradation pathway. When this is not achieved, intranuclear inclusions are formed. These inclusions trigger neurodegeneration by activation of neurotoxic signaling pathways.

Although this syndrome was associated exclusively with carriers of premutation alleles, some cases of FXTAS among carriers of intermediate alleles have recently been published [106,107]. It has also been observed in individuals with full mutation and lack of methylation or mosaicism. FXTAS can also be present among females carrying premutation alleles, though with a less severe phenotype [108].

## 4. Fragile X-Associated Primary Ovarian Insufficiency (FXPOI)

Cronister et al. in 1991 [109] studied the correlation between *FMR1* gene and POI. They found that 13% of carrier women developed menopause before the age of 40. This percentage was significantly higher than in controls (5%). The identification of the *FMR1* gene in 1991 and the development of diagnostic techniques allowed the analysis and differentiation of *FMR1* alleles. Since 1994, evidence has shown that women carrying *FMR1* premutation and intermediate alleles have a risk of developing POI. Women with full mutation alleles, instead, have the same risk for POI as the general population (1%).

### 4.1. POI and Premutation Alleles

Schwartz et al. [110] were the first to indicate that premutation carriers have a higher risk for POI than full mutation carriers. In 1999 Allingham-Hawkins et al. [111] determined in an international study the prevalence of POI among women carriers or non-carriers of premutation and full mutation alleles. They estimated that 16% of women carrying premutation alleles had POI. Other authors have confirmed this prevalence [112,113,114,115,116,117,118].

Therefore, only a percentage of women with premutation alleles suffer ovarian dysfunction. The identification of factors influencing clinical variability and that help to identify those women at risk of reproductive complications is important.

Concerning the molecular level, a non-linear association between premutation repeat size and risk for ovarian failure has been found [116,119,120], as those with premutation sizes of ~80–100 repeats have a higher risk for POI and earlier age of menopause. Additionally, some authors have shown that premutation carriers without signs of ovarian dysfunction have a reduction in the average age at menopause compared with non-carriers [113,121,122]. Sullivan et al. [116] established this reduction as five years. In addition, higher levels of FSH, an indicator of reduced ovarian function, were found in premutation carriers still cycling [123,124,125].

### 4.2. Premutation Alleles among Women with POI

Once the prevalence of POI among premutation carrier women was established, some studies were carried out to learn the frequency of premutation alleles among women with POI of unknown etiology. Conway et al. [17] were the first to perform an analysis in women with idiopathic POI. They found that the frequency of premutation alleles among women with sporadic POI and familial POI was 1.6% and 16% respectively.

After the study of Conway et al. other authors examined groups of women with POI and the estimation of premutation carriers has been established at 3.2% and 11.5% among women with sporadic or familial POI respectively [1,126].

Consequently, among women carrying FMR1 premutation alleles there is a continuum of diminished ovarian reserve that give rise to menstrual cycle irregularities, decreased fertility and hormone fluctuations. To involve all these traits, Welt [7] recommended the use of the term fragile X primary ovarian insufficiency (FXPOI) for the cases in which ovarian dysfunction was associated to FMR1 gene.

### 4.3. POI and Intermediate Alleles

POI has also been related to *FMR1* intermediate alleles [127,128]. Bretherick et al. [127] found that 14.2% of alleles among women with POI had between and including 35 and 54 repeats. Bodega et al. [128] proposed that this relation could depend on the AGG interspersion pattern.

Pastore et al. [129] indicated that alleles between 35 and 44 CGGs were overrepresented among women with diminished ovarian function but regular menses. Streuli et al. [118] also found a higher percentage of alleles >40 CGGs in women with occult primary ovarian insufficiency. Karimov et al. [130] published similar results.

On the other hand, authors such as Bennett et al. [131], Murray et al. [132] or Voourhis et al. [133] did not find an association between *FMR1* intermediate alleles and primary ovarian insufficiency.

### 4.4. Molecular and Cellular Mechanisms

Molecular mechanisms that compromise follicular function are unknown but it is proposed that they can occur at various stages of follicular development and could be related to: an initial decrease in the ovarian reserve, altered recruitment of the follicle, increased atresia, or altered hypothalamic-pituitary-gonadal axis. FMRP is expressed in different areas that could affect these processes such as the brain and critic regions for hormonal regulation.

An important question is why the ovarian dysfunction is limited to premutation and intermediate carrier women, and it is not observed in full mutation carriers. The *FMR1*-mRNA levels are higher in premutation and intermediate carriers, so a direct or indirect effect of mRNA levels has been proposed, similar to that which occurs in FXTAS. Conway et al. [17] suggested a toxic effect of the elevated levels of *FMR1*-mRNA that can lead to a diminished ovarian reserve before birth. Later, Sullivan et al. [116] proposed that ovarian dysfunction associated to premutation and intermediate alleles could be considered a late age-of-onset disorder. The mRNA toxic effect leads to increased atresia or apoptosis of follicles.

Among female premutation carriers *FMR1*-mRNA levels do not seem to be parallel to the increase in CGG repeats [57,134,135]. This could be related with the non-linear association between premutation repeat size and risk for ovarian failure proposed previously by Ennis et al. [120].

Lu et al. [136] analyzed a premutation carrier mouse and observed that the mRNA increase was sufficient to cause a reduction in the follicle number leading to a deficient fertility. However, Hoffman et al. [137] found that development and establishment of primordial follicle pool was normal but there was an accelerated loss of follicles, suggesting an intrinsic problem of the ovary.

Investigations in humans have noted that FMRP is expressed in oocytes of fetal ovaries [138] and in granulose cells of mature follicles [135]. More recently, it has been shown that *FMR1*-mRNA levels are elevated in granulose cells and that these levels have not a lineal association with CGG repeats [139].

Some authors [135,140,141] consider FMRP and its expression in granulose cells a quantitative trait that controls ovarian reserve: during follicular maturation granulose cells express some genetically controlled endocrine signals and signaling pathways important for the correct maturation of oocytes. These signals could be reduced or disappear when *FMR1*-mRNA and FMRP (which act as translational repressors by binding to multiple transcripts and controlling their level of translation) are altered among premutation and intermediate carriers.

Therefore, elevated levels of *FMR1*-mRNA observed in women carrying intermediate and premutation alleles could give rise to an elevated toxicity that would lead to a higher atresia of the follicles [34] or to a reduction in the number of growing follicles like in the mouse model. In both cases the result would be diminished fertility.

## 5. Summary

*FMR1* gene is one of the most important genes related to POI. For this reason, it is important to analyze *FMR1* gene among women presenting signs of ovarian dysfunction of unknown cause. This analysis must include the study of *FMR1* gene structure, including both CGG repeat number and AGG interruption pattern. This would be useful for knowing not only the etiology of ovarian dysfunction but also for detecting intermediate and premutation alleles that could expand in some generations leading to a mutation and consequently to a fragile X syndrome. Moreover, detection of these alleles among young women allows the genetic counseling necessary for planning their reproductive lives considering possible ovarian dysfunction. In relation to the molecular mechanisms, more research in humans is needed to elucidate how *FMR1*-mRNA and FMRP act in the ovaries.

## Figures and Tables

**Table 1:** Genes that have been associated with primary ovarian insufficiency (POI). Adopted and modified from Qin et al. [15].

Gene	Localization
*BMP15*—*Bone morphogenetic protein 15*	Xp11.22
*AR*—*Androgen receptor*	Xq12
*FOXO4*—*Forkhead box O4*	Xq13.1
*POF1B*—*Premature ovarian failure, 1B*	Xq21.2
*DACH2*—*Dachshund family transcription factor 2*	Xq21.3
*PGRMC1*—*Progesterone receptor membrane component 1*	Xq22-q24
*FMR1*—*Fragile X mental retardation 1*	Xq27.3
*FMR2*—*Fragile X mental retardation 2*	Xq28
*FIGLA*—*Folliculogenesis specific bHLH transcription factor*	2p13.3
*LHR*—*Luteinizing hormone receptor*	2p21
*FSHR*—*Follicle-stimulating hormone receptor*	2p21-p16
*INHA*—*Inhibin A*	2q35
*GDF9*—*Growth differentiation factor 9*	5q31.1
*FOXO3a*—*Forkhead box O3*	6q21
*NOBOX*—*Newborn ovary homeobox gene*	7q35
*STAG3*—*Stromal Antigen 3*	7q22.1
*AMHR2*—*Anti-Mullerian hormone receptor, type II*	12q13
*FOXO1*—*Forkhead box O1*	13q14.1
*SPO11*—*Meiotic protein covalently bound to DSB*	20q13.31
*DMC1*—*DNA meiotic recombinase 1*	22q13.1
